# Improving Diagnostic Classification of Stillbirths and Neonatal Deaths Using ICD-PM (International Classification of Diseases for Perinatal Mortality) Codes: Validation Study

**DOI:** 10.2196/20071

**Published:** 2020-08-03

**Authors:** Hiu Mei Luk, Emma Allanson, Wai-Kit Ming, Wing Cheong Leung

**Affiliations:** 1 Department of Obstetrics and Gynaecology Kwong Wah Hospital Hong Kong SAR China (Hong Kong); 2 Institute of Health Research University of Notre Dame, Fremantle Western Australia Australia; 3 Department of Public Health and Preventive Medicine School of Medicine Jinan University Guangzhou China

**Keywords:** stillbirths, perinatal deaths, neonatal deaths, ICD-PM, ICD-10

## Abstract

**Background:**

Stillbirths and neonatal deaths have long been imperfectly classified and recorded worldwide. In Hong Kong, the current code system is deficient (>90% cases with unknown causes) in providing the diagnoses of perinatal mortality cases.

**Objective:**

The objective of this study was to apply the International Classification of Diseases for Perinatal Mortality (ICD-PM) system to existing perinatal death data. Further, the aim was to assess whether there was any change in the classifications of perinatal deaths compared with the existing classification system and identify any areas in which future interventions can be made.

**Methods:**

We applied the ICD-PM (with International Statistical Classification of Diseases and Related Health Problems, 10th Revision) code system to existing perinatal death data in Kwong Wah Hospital, Hong Kong, to improve diagnostic classification. The study included stillbirths (after 24 weeks gestation) and neonatal deaths (from birth to 28 days). The retrospective data (5 years) from May 1, 2012, to April 30, 2017, were recoded by the principal investigator (HML) applying the ICD-PM, then validated by an overseas expert (EA) after she reviewed the detailed case summaries. The prospective application of ICD-PM from May 1, 2017, to April 30, 2019, was performed during the monthly multidisciplinary perinatal meetings and then also validated by EA for agreement.

**Results:**

We analyzed the data of 34,920 deliveries, and 119 cases were included for analysis (92 stillbirths and 27 neonatal deaths). The overall agreement with EA of our codes using the ICD-PM was 93.2% (111/119); 92% (78/85) for the 5 years of retrospective codes and 97% (33/34) for the 2 years of prospective codes (*P*=.44). After the application of the ICD-PM, the overall proportion of unknown causes of perinatal mortality dropped from 34.5% (41/119) to 10.1% (12/119) of cases (*P*<.001).

**Conclusions:**

Using the ICD-PM would lead to a better classification of perinatal deaths, reduce the proportion of unknown diagnoses, and clearly link the maternal conditions with these perinatal deaths.

## Introduction

### Background

More than 5 million perinatal deaths occur globally each year, and this largely silent epidemic significantly impacts and burdens families [1,2]. Despite this, perinatal deaths have long been frequently invisible and poorly recorded worldwide.

A large proportion of stillbirth and neonatal death cases take place in less developed countries [3]. Classification methods used in these countries can be obscure. The numbers of skilled medical personnel are often inadequate, which contributes to less than comprehensive recording of clinical data at the time of stillbirth and neonatal deaths [4,5]. In contrast, stillbirth affects proportionally fewer births in high-income countries, and data related to these deaths tend to be comprehensively recorded [6]. Many high-income countries have developed their own classification systems for perinatal deaths; for example, the United Kingdom uses the Codac system [7], Sweden has the Stockholm system [8], Australia and New Zealand use the Perinatal Society of Australia and New Zealand perinatal death classification system [9], and the Netherlands uses the Tulip classification system [10]. In the United States, the Stillbirth Collaborative Research Network developed the initial causes of fetal death system [11]. While we see an annual reduction in the rate in stillbirth globally [12], this trend differs widely among high- and low- income countries. An internationally recognized classification system of perinatal mortality would be invaluable so that the data could easily be compared between different countries to facilitate classification and research in driving programs to reduce the overall perinatal mortality.

Based on the *International Statistical Classification of Diseases and Related Health Problems, 10th Revision* (ICD-10), the International Classification of Diseases for Perinatal Mortality (ICD-PM) was released by the World Health Organization in August 2016 [13]. It is the first perinatal death classification system developed to be used worldwide. This system links the cause of perinatal death, using ICD-10 codes [14], separated by timing of death, with the maternal conditions that contribute to perinatal death.

Currently, in Hong Kong under the Hospital Authority, the coding system (for stillbirths only) used [15] is a direct one (ie, if the cause of the stillbirth could be identified, it would be stated directly such as congenital abnormalities, pregnancy-induced hypertension, cord accident, antepartum hemorrhage, maternal disease). The remaining cases would be placed in the categories unclassified, unexplained, and miscellaneous/uninvestigated, which essentially refer to unknown causes. The problem is that up to 92.8% (800/862) of stillbirths from 2012 to 2018 were classified under these 3 categories [15]. This phenomenon has significantly impaired the potential of using this perinatal database to further study and design programs to reduce perinatal mortality.

We hypothesized that by using a globally applicable classification system such as ICD-PM that recognizes stillbirths and neonatal deaths together with the contributing maternal conditions, coding of stillbirth and neonatal death could be significantly improved. Therefore, a validation study was performed to apply the ICD-PM coding system to our stillbirths and neonatal death cases.

### Outcome

The primary outcome was the reduction in unknown causes for stillbirth and neonatal death after applying the ICD-PM coding system. The secondary outcome was the percentage agreement with expert (EA) in applying ICD-PM codes.

## Methods

### Recruitment

The ICD-PM system, the World Health Organization application of the ICD-10 to deaths during the perinatal period, was applied to existing perinatal death data from May 1, 2012, to April 30, 2019, in Kwong Wah Hospital, a regional public hospital with 34,920 deliveries during the 7-year study period.

### Selection Criteria

Inclusion criteria:

- Stillbirth cases diagnosed after 24 completed weeks gestation
- Neonatal death cases within 28 days of birth

Exclusion criteria:

- Miscarriage at less than 24 completed weeks of gestation
- Termination of pregnancy due to fetal anomalies or maternal abnormal medical conditions

### Ethics

Ethics approval for the study was granted by the Kowloon Central/Kowloon East Research Ethics Committee (KC/KE-19-0193/ER-1). As this clinical study did not involve active patient participation, no patient consent was required.

### Diagnostic Classification

In our department, every case of stillbirth and neonatal death is discussed in our monthly perinatal meetings attended by consultants, specialists, trainees, and senior labor ward midwives and senior nurses from the departments of obstetrics and gynecology and pediatrics. A detailed case summary is presented by the resident trainee directly involved in the clinical management of that particular case. Diagnosis of the cause of stillbirth and neonatal death is based on the clinical findings and investigation results.

We performed routine investigations for stillbirths, including:

- Maternal: complete blood counts; liver and renal function tests; urate; clotting profile; thyroid function test; lupus anticoagulant; anticardiolipin antibodies; antinuclear antibodies +/– anti-ds DNA; rheumatoid factor; Kleihauer test; toxoplasmosis, rubella, cytomegalovirus, and herpes simplex virus tests; oral glucose tolerance test; hemoglobin A1c; high vaginal swab; midstream urine for culture; and others.
- Placenta: swabs for culture, histopathology, karyotyping (if the stillbirth showed dysmorphic features)
- Stillbirth: body surface swabs for culture, autopsy (with consent from parents)

### Data Validation

Our validation study started in 2017 and was divided into two parts.

#### Retrospective Validation Study (Five Years)

The recoding of the retrospective stillbirth and neonatal death data from May 1, 2012, to April 30, 2017, was performed by the principal investigator (HML) using the ICD-10 [14] to get a specific ICD-10 code that was then converted to the ICD-PM categories [13].

Our ICD-10 and ICD-PM codes together with the detailed case summaries for each stillbirth and neonatal death case were then forwarded to an overseas expert (EA; via emails without patient identity) for validation of our codes based on the detailed case summaries, further discussion, and verification. EA has extensive experience using the ICD-PM [1-4]. The proportion of ICD-PM codes EA disagreed with was noted.

#### Prospective Validation Study (Two Years)

The prospective application of ICD-10 and then ICD-PM was performed from May 1, 2017, to April 30, 2019. The coding for each stillbirth and neonatal death case was validated during our monthly perinatal meetings.

Our ICD-PM codes together with the detailed case summaries in this prospective case series were also forwarded to the overseas expert (EA) to be checked for agreement.

### Statistical Analysis

All frequency data were analyzed by summary statistics. SPSS Statistics version 25.0 (IBM Corporation) was used for the analysis. The Pearson chi-square test was used where appropriate, such as to determine whether there was a significant difference between the frequency of unknown causes of death classified in our original Hospital Authority coding system and the newly applied ICD-PM coding system. *P*<.05 was considered statistically significant.

## Results

We analyzed data for 34,920 deliveries, and 119 cases were included for analysis (92 stillbirths and 27 neonatal deaths):

- Retrospective ICD-PM codes (5 years; May 1, 2012, to April 30, 2017)—total 85 cases
- Prospective ICD-PM codes (2 years; May 1, 2017, to April 30, 2019)—total 34 cases

All stillbirth cases had the full set of routine investigations described under Methods. However, only 25.2% (30/119) of cases had a postmortem examination.

EA verified every single one of the 119 stillbirth and neonatal death cases during the study period. The overall agreement rate of our codes using ICD-10 and then ICD-PM was 93.2% (111/119 cases) with EA: 92% (78/85 cases) for the 5 years of retrospective cases and 97% (33/34 cases) for the 2 years of prospective cases (*P*=.44). It was interesting and educational to look at how EA disagreed with our codes (Table 1).

Table 2 illustrates the application of the ICD-PM for perinatal death and maternal condition in stillbirth and neonatal death cases, respectively. In the ICD-PM, there are 6 groups of antepartum causes for stillbirth (A1 to A6), 7 groups of intrapartum causes for stillbirth (I1 to I7), 11 groups of causes for neonatal death (N1 to N11), and 5 groups of maternal conditions (M1 to M5) to be associated with stillbirth or neonatal death.

The most common causes for antepartum stillbirths were A3 (antepartum hypoxia, 24/91, 26%), followed by A5 (disorders related to fetal growth, 17/91, 19%), and A1 (congenital malformations, deformations, and chromosomal abnormalities, 10/91, 11%). In this case series, there was only one intrapartum stillbirth (I1). The most common corresponding maternal conditions were M1 (complications of placenta, cord, and membranes, 39/91, 43%), followed by M4 (maternal medical and surgical conditions, 21/91, 23%), and M2 (maternal complications of pregnancy, 12/91, 13%). The most common associations were A3;M1 (20/91, 22%), A6;M5 (12/91, 13%), and A6;M4 (9/91, 10%).

On the other hand, the most common causes for neonatal deaths were N9 (low birth weight and prematurity, 9/27, 33%), followed by N8 (neonatal conditions, 5/27, 19%), N6 (infection, 4/27, 15%), and N1 (congenital malformations, deformations and chromosomal abnormalities, also 4/27, 15%). The most common corresponding maternal conditions were M1 (complications of placenta, cord, and membranes, 10/27, 37%), followed by M2 (maternal complications of pregnancy, 6/27, 22%) and M4 (maternal medical and surgical conditions, 5/27, 19%). The most common associations were N9;M1 (3/27, 11%), N9;M4 (3/27, 11%), and N6; M1 (3/27, 11%).

**Table 1:** The 8 cases in which the overseas expert (EA) disagreed with our codes.

Case number	Our original codes	Our ICD-PM^a^ codes	EA’s codes	Comment
1	Unknown	A6; M1	A6; M5	EA: wonder if the placental pathology showing chorioamnionitis (M1) is related to the stillbirth in the absence of other evidence
9	Preeclampsia with placenta abruptio	A3; M2	A3; M4	EA: would classify this as M4 (PET^b^) as the fetus died before the mother, likely as a result of the PET, rather than the maternal death (M2) being the cause of the fetal death
11	Twin-twin transfusion syndrome (TTTS^c^), postmortem–hypoplastic adrenals	A1, M1	A1, M5	EA: Do you think the hypoplastic adrenals (A1) was the cause of death? Or hypoxia as a result of the TTTS (M1) with the hypoplastic adrenals being a secondary issue?
19	Unknown	A6; M1	A6; M5	EA: As long as you are certain of the chorioamnionitis (M1) again, or is this a postmortem change (M5) between fetal death and delivery?
23	Unknown	A5; M4	A6; M4	EA: The birthweight is surely to be expected with the delay between fetal death and delivery, and there is no evidence of IUGR^d^ (A5); keep M4 (GDM^e^).
31	Unknown	A6; M1	A6; M5	EA: Are you confident of the chorioamnionitis (M1) as the cause of death?
71	Fetal syndromal abnormality	A1; M3	A1; M5	EA: The cesarean delivery was done after the fetal death. Cesarean delivery as a cause is more when there are complications (M3) from the cesarean delivery that lead to the fetal death
89	Cord accident, drug addict	A3; M4	A3; M1	EA: Cord accident (A3; M1) as the cause of fetal death rather than mother is drug addict (M4)

^a^ICD-PM: International Classification of Disease for Perinatal Mortality.

^b^PET: pre-eclampsia.

^c^TTTS: twin-twin transfusion syndrome.

^d^IUGR: intrauterine growth restriction.

^e^GDM: gestational diabetes mellitus.

**Table 2:** ICD-PM codes for stillbirths (antepartum [A] and intrapartum [I]) and neonatal [N] deaths with maternal conditions (n=119).

Perinatal cause of death		Maternal condition					
		M1: complications of placenta, cord, and membrane	M2: maternal complications of pregnancy	M3: other complications of labor and delivery	M4: maternal medical and surgical conditions	M5: no maternal conditions	Total (%)
Antenatal death (A)							
	A1: congenital malformations, deformations, and chromosomal abnormalities	2	2	0	2	4	10 (11.0)
	A2: infection	6	0	0	0	1	7 (7.7)
	A3: antepartum hypoxia	20	1	0	3	0	24 (26.4)
	A4: other specified antepartum disorder	1	0	0	1	0	2 (2.2)
	A5: disorders related to fetal growth	6	5	0	6	0	17 (18.7)
	A6: fetal death of unspecified cause	4	4	2	9	12	31 (34.0)
	Total (%)	39 (42.9)	12 (13.2)	2 (2.2)	21 (23.0)	17 (18.7)	91 (100.0)
Intrapartum death (I)							
	I1: congenital malformations, deformations, and chromosomal abnormalities	0	0	1	0	0	1 (100.0)
	I2: birth trauma	0	0	0	0	0	0 (0.0)
	I3: acute intrapartum event	0	0	0	0	0	0 (0.0)
	I4: infection	0	0	0	0	0	0 (0.0)
	I5: other specified intrapartum disorder	0	0	0	0	0	0 (0.0)
	I6: disorders related to fetal growth	0	0	0	0	0	0 (0.0)
	I7: intrapartum death of unspecified cause	0	0	0	0	0	0 (0.0)
	Total (%)	0	0	1 (100.0)	0	0	1 (100.0)
Neonatal death (N)							
	N1: congenital malformations, deformations, and chromosomal abnormalities	1	1	2	0	0	4 (14.8)
	N2: disorders related to fetal growth	0	0	0	1	0	1 (3.7)
	N3: birth trauma	0	0	0	0	0	0 (0.0)
	N4: complications of intrapartum events	1	0	0	0	0	1 (3.7)
	N5: convulsions and disorders of cerebral status	0	0	0	0	0	0 (0.0)
	N6: infection	3	1	0	0	0	4 (14.8)
	N7: respiratory and cardiovascular disorders	0	2	1	0	0	3 (11.1)
	N8: neonatal conditions	2	1	0	1	1	5 (18.5)
	N9: low birth weight and prematurity	3	1	1	3	1	9 (33.4)
	N10: miscellaneous	0	0	0	0	0	0 (0.0)
	N11: neonatal death of unspecified cause	0	0	0	0	0	0 (0.0)
	Total (%)	10 (37.1)	6 (22.2)	4 (14.8)	5 (18.5)	2 (7.4)	27 (100.0)

Before the application of the ICD-PM coding system, 34.5% (41/119) of stillbirths and neonatal deaths were classified as having unknown causes. After the application of the ICD-PM system, the cases with unknown causes of perinatal death dropped to 10.1% (12/119) cases (*P*<.001). In our study, all neonatal deaths had specific causes identified.

After retrospectively applying ICD-10 and ICD-PM codes in the cases with unknown causes (30/85, 35%) of stillbirth from our original classification on the 85 cases from 2012 to 2017 (retrospective validation study), we can further reduce the unknown causes (ie, A6;M5) to 8% (7/85) of cases (*P*<.001). Table 3 showed how we could change the 23 unknown (30 minus 7) to known causes using ICD-PM.

**Table 3:** In the retrospective 5-year validation study (May 1, 2012, to April 30, 2017), 23/30 unknown causes of stillbirth can be assigned a diagnosis category after applying ICD-PM code.

Case number	Original codes	Remarks	ICD-PM^a^ codes
3	Unknown	Funisitis, GDM^b^	A2; M1
8	Unknown	Maternal hypothyroidism	A6; M4
14	Unknown	History of deep vein thrombosis, placental histopathology: fetal thrombotic vasculopathy and placenta infarction	A3; M1
23	Unknown	480 g at 28+ weeks, GDM	A6; M4
28	Unknown	375 g at 24+ weeks, placenta: thrombotic vasculopathy	A5; M1
32	Unknown	Placenta: focal fetal thrombotic vasculopathy	A6; M1
39	Unknown	GDM	A6; M4
48	Unknown	GDM	A6; M4
50	Unknown	2520 g at 39 weeks; placenta: thrombotic vasculopathy	A5; M1
55	Unknown	400 g at 25 weeks; severe oligohydramnios	A5; M2
57	Unknown	Preeclampsia, DM^c^ in pregnancy	A6; M4
58	Unknown	Polyhydramnios	A6; M2
59	Unknown	250 g at 25 weeks; twisted cord	A5; M1
60	Unknown	255 g at 24 weeks; GDM	A5; M4
61	Unknown	330 g at 24 weeks; GDM	A5; M4
62	Unknown	Breech presentation	A6; M3
65	Unknown	Twin pregnancy	A6; M2
66	Unknown	Twin pregnancy	A6; M2
72	Unknown	Placenta: chorioamnionitis (Escherichia coli); fetal thrombotic vasculopathy	A2; M1
73	Unknown	Placenta: fetal thrombotic vasculopathy	A6; M1
74	Unknown	Oligohydramnios	A6; M2
80	Unknown	Maternal infection with fever; placenta: focal intervillous thrombus	A2; M1
85	Unknown	Placenta: minor infarcted villi	A6; M1

^a^ICD-PM: International Classification of Diseases for Perinatal Mortality.

^b^GDM: gestational diabetes mellitus.

^c^DM: diabetes mellitus.

## Discussion

### Principal Findings

Perinatal deaths remain problematic worldwide. Before the advent of the ICD-PM, there were several independent systems in high-income countries and few systems in low- and middle- income countries, causing significant disparity in perinatal death data recorded among countries [16]. The classification system used in Hong Kong Hospital Authority obstetrics units is no better, with 92.8% of cases having unknown causes [15]. It seems that we were somehow reluctant to give a specific diagnosis under the original coding system unless the cause was crystal clear. Hence detailed and accurate information that can be retrieved from our original classification of perinatal mortality cases was scarce. The ICD-PM is the first system that addresses the issue internationally. ICD-10 and ICD-PM are user-friendly as shown by the high level of agreement (93.2% overall) between our codes with that by the international expert (EA). The agreement with EA of our codes using ICD-PM was even higher, reaching 97.1% for the 2 years of prospective cases compared with 91.8% for the 5 years of retrospective cases, although not statistically significant (*P*=.44). This further supports our observation that the final consensus code made during perinatal meetings with multidisciplinary discussion on the perinatal death cases gives the most accurate answer, although our principal investigator (HML) had already done a good job with the 5-year retrospective codes.

The ICD-PM is designed to be used for classifying stillbirths and neonatal deaths at three levels. First, it identifies the time of death as antepartum (before the onset of labor), intrapartum (during labor but before delivery), or neonatal (up to day 7 of postnatal life, can be extended to late neonatal deaths, so as within 28 days of life, like in our classification). Second, it is multilayered such that the depth of classification can reflect the locally available intensity of investigation. In our Hospital Authority obstetrics units, the investigations for stillbirths and neonatal deaths are quite in-depth, as described under Methods, but this was not be reflected by the original classification system. Third, the ICD-PM links the contributing maternal condition, if any, with perinatal death. This is very important because most of these contributing maternal conditions would not have shown up in our original coding system. Ultimately, the ICD-PM classification at all three levels allows easy identification of where a program intervention should be targeted in order to improve the perinatal outcomes.

After the application of the ICD-PM system, the ratio of unknown causes of perinatal mortality dropped from 34.5% to 10.1%, which was statistically significant (*P*<.001). A total of 77% of stillbirth cases initially classified as unknown were now assigned a diagnostic category after applying the ICD-PM (Table 3). In our study, all neonatal deaths had specific causes identified. In other words, the ICD-PM is more useful for coding stillbirths than neonatal deaths in our locality.

The main benefit of using ICD-10 and ICD-PM codes was to have a better understanding of the perinatal deaths in terms of the timing of death, the depth of investigations, and any contributing maternal conditions. We also consider the ICD-PM code to be user-friendly for changing an existing local perinatal death classification to one that is global and can be compared with international data. Using the ICD-PM code can lead to better classification of perinatal deaths, reduce the proportion of unknown diagnoses, and clearly link the maternal conditions with these perinatal deaths. There are some countries such as the United Kingdom [3], South Africa [3,16,17], India [18], and Colombia [19] using the ICD-PM to interpret the perinatal mortality data which showed that the ICD-PM classification was feasible and enabling the characterization of perinatal mortality. This is the first validation study demonstrating the application of the ICD-PM coding system in Hong Kong.

### Limitations

However, there are limitations to our study. The study was focused on one hospital only, so the sample size was small. The improvement in coding might be related to the enthusiastic principal investigator (HML) as the coder in the retrospective validation study; the code in the prospective validation study was done during our monthly perinatal meetings with multidisciplinary input from various stakeholders, which is our usual practice before and after the study. Whether there is an overall Hawthorne effect (improving performance of coding during the study period) could be seen by continuous monitoring of the diagnostic classification of stillbirths and neonatal deaths after the ICD-PM is formally used for coding from 2020 onward.

Further study can be performed in all Hong Kong Hospital Authority obstetrics units using the ICD-10 and ICD-PM so as to draw an overall picture of perinatal mortality across the territory.

### Conclusions

The ICD-PM is a user-friendly system that can enhance the understanding of data [5]. Using the ICD-PM coding system could lead to a more comprehensive classification of perinatal deaths, reduce the proportion of unknown causes as well as providing better linkage to maternal conditions in these perinatal deaths. The ICD-PM classifications are more extensive in covering diagnostic categories, with more specific details. Implementing this new coding system in Hong Kong Hospital Authority obstetrics units will be of great help in improving clinical practice and reducing perinatal mortality in the long run.

## Abbreviations

ICD-10: International Statistical Classification of Diseases and Related Health Problems, 10th Revision
ICD-PM: International Classification of Diseases for Perinatal Mortality
